# Pain sensitivity as a state marker and predictor for adolescent non-suicidal self-injury

**DOI:** 10.1017/S0033291724000461

**Published:** 2024-07

**Authors:** Han-Tin Kao, Ines Mürner-Lavanchy, Elisabeth von Stosch, Johannes Josi, Thomas Berger, Julian Koenig, Michael Kaess

**Affiliations:** 1 University Hospital of Child and Adolescent Psychiatry and Psychotherapy, University of Bern, Bern, Switzerland; 2 Section for Experimental Child and Adolescent Psychiatry, Department of Child and Adolescent Psychiatry, Centre for Psychosocial Medicine, Heidelberg University, Heidelberg, Germany; 3 Department of Clinical Psychology and Psychotherapy, University of Bern, Bern, Switzerland; 4 Faculty of Medicine and University Hospital Cologne, Department of Child and Adolescent Psychiatry, Psychosomatics and Psychotherapy, University of Cologne, Cologne, Germany; 5 Section for Translational Psychobiology in Child and Adolescent Psychiatry, Department of Child and Adolescent Psychiatry, Centre for Psychosocial Medicine, Heidelberg University, Heidelberg, Germany

**Keywords:** adolescents, borderline personality disorder, longitudinal study, non-suicidal self-injury, pain sensitivity

## Abstract

**Background:**

The pain analgesia hypothesis suggests that reduced pain sensitivity (PS) is a specific risk factor for the engagement in non-suicidal self-injury (NSSI). Consistent with this, several studies found reduced PS in adults as well as adolescents with NSSI. Cross-sectional studies in adults with borderline personality disorder (BPD) suggest that PS may (partially) normalize after remission or reduction of BPD symptoms. The objective of the present study was to investigate the development of PS over 1 year in a sample of adolescents with NSSI and to investigate whether PS at baseline predicts longitudinal change in NSSI.

**Methods:**

*N* = 66 adolescents who underwent specialized treatment for NSSI disorder participated in baseline and 1-year follow-up assessments, including heat pain stimulation for the measurement of pain threshold and tolerance. Associations between PS and NSSI as well as BPD and depressive symptoms were examined using negative binomial, logistic, and linear regression analyses.

**Results:**

We found that a decrease in pain threshold over time was associated with reduced NSSI (incident rate ratio = 2.04, *p* = 0.047) and that higher pain tolerance at baseline predicted lower probability for NSSI (odds ratio = 0.42, *p* = 0.016) 1 year later. However, the latter effect did not survive Holm correction (*p* = 0.059). No associations between PS and BPD or depressive symptoms were observed.

**Conclusion:**

Our findings suggest that pain threshold might normalize with a decrease in NSSI frequency and could thus serve as a state marker for NSSI.

## Introduction

The pain analgesia hypothesis suggests that individuals who engage in self-injury experience less pain and are thus less averse to injure themselves (Nock, 2010). This reduced pain sensitivity (PS) is seen as a specific risk factor for self-injury. The habituation hypothesis also suggests that reduced PS and non-suicidal self-injury (NSSI) are associated but proposes that reduced PS in NSSI develops over time via habituation to repeated self-injury (Nock, Joiner, Gordon, Lloyd-Richardson, & Prinstein, 2006). Consistent with these hypotheses, altered PS has been demonstrated in individuals engaging in NSSI (Glenn, Michel, Franklin, Hooley, & Nock, 2014; Koenig et al., 2017b; Koenig, Thayer, & Kaess, 2016), but also in borderline personality disorder (BPD) (Koenig et al., 2016; Ludäscher et al., 2015; Van der Venne et al., 2021) and depression (Thompson, Correll, Gallop, Vancampfort, & Stubbs, 2016), two disorders that are often comorbid to NSSI (Ghinea et al., 2020). Standard measures of experimental PS include *pain threshold* (the minimum stimulus intensity or time of exposure required to produce the sensation of pain) and *pain tolerance* (the maximum stimulus intensity or time of exposure an individual is willing to tolerate) (Bartley & Fillingim, 2013), with greater PS being reflective of lower pain threshold and tolerance.

A meta-analysis by Koenig et al. (2016) revealed greater pain threshold and tolerance as well as less self-reported pain intensity in individuals with self-harm (i.e. including patients with NSSI and BPD) compared to healthy controls (HC). Cross-sectional findings in adults with BPD suggest that PS may (partially) normalize after remission or reduction of BPD symptoms (Bekrater-Bodmann et al., 2015; Ludäscher et al., 2009). Bekrater-Bodmann et al. (2015) showed that remitted BPD patients displayed normalized heat pain thresholds similar to those of HC. Interestingly, Ludäscher et al. (2009) showed that patients with BPD who currently injured themselves demonstrated lower pain thresholds than patients with BPD who stopped injuring themselves. The only longitudinal study including adolescents engaging in NSSI did not find an association between change in pain threshold and reduction in NSSI after a 1-year follow-up (FU) (Koenig et al., 2017a). In contrast to that, greater NSSI reduction was associated with increased pain tolerance, which suggests that PS does not normalize after reduction of NSSI. Instead, NSSI may be terminated once pain tolerance gets too high since the pain stimulus is no longer perceived as aversive enough to effectively relieve stress.

A meta-analysis by Thompson et al. (2016) demonstrated higher pain thresholds in adult patients with a depressive disorder compared to HC for low-intensity pain stimulation. In line with this, a review by Kim et al. (2022) demonstrated that adult patients with a depressive disorder showed increased pain thresholds compared to HC across several pain modalities. In contrast, the associations between pain tolerance and intensity with depression were inconsistent. So far, findings on altered PS in depression have not been replicated in adolescents. Investigating the relationship between PS and depressive symptoms in adolescents with NSSI, Van der Venne et al. (2021) found lower pain intensity to be associated with higher depressive symptom scores, but no association between pain threshold and tolerance and depressive symptoms was found.

As mentioned above, Koenig et al. (2017a) found no longitudinal association between changes in PS and NSSI in adolescents. However, this study was likely underpowered due to a small sample size. Here, we aimed to investigate this association in a large sample of adolescents. Furthermore, we wanted to extend these findings by investigating whether PS can serve as a predictor for improvement in clinical symptoms possibly attributed to psychotherapy. To our knowledge, this has not been studied so far. Several studies have demonstrated that higher symptom severity and lower levels of functioning predict poorer therapy response across different psychiatric disorders and types of treatments (Hoeboer et al., 2021; Jarrett et al., 2013; Lindfors, 2022; Sotsky et al., 1991). As such, higher levels of self-criticism (Löw, Schauenburg, & Dinger, 2020), self-blame, and emotion suppression (Ryum, Vogel, Walderhaug, & Stiles, 2015; Scherer, Boecker, Pawelzik, Gauggel, & Forkmann, 2017) have been shown to predict poorer therapy outcome. This is of interest since lower PS was associated with higher symptom severity (Ludäscher et al., 2009; Van der Venne et al., 2021), emotion dysregulation, and self-criticism (Bunderla & Kumperščak, 2015) as well as self-punishment (Hamza, Willoughby, & Armiento, 2014). On the contrary, there are also studies that did not find an association between PS and overall psychopathology (Glenn et al., 2014; Ludäscher et al., 2015) or symptom severity (Van der Venne et al., 2021).

The current study aimed to investigate

1. The longitudinal covariance of PS with NSSI frequency (and with BPD as well as depressive symptoms) over 1 year in adolescents undergoing specialized treatment for NSSI
2. Whether PS at baseline (BL) predicts change in NSSI frequency (and in BPD as well as depressive symptoms) over 1 year in adolescents with NSSI.

We defined NSSI as our primary outcome and BPD symptoms as well as depressive symptoms as our secondary outcomes. Accordingly, we divided our hypotheses in main hypotheses with NSSI as outcome and secondary hypotheses with BPD/depressive symptoms as outcomes. Based on cross-sectional findings in adults with BPD (Bekrater-Bodmann et al., 2015; Ludäscher et al., 2009) our first main hypothesis H1a is that an increase in PS (i.e. decrease in pain threshold and in pain tolerance) is associated with a reduction in NSSI. In accordance with the conceptualization of PS as a risk factor and some studies that suggest lower PS to reflect higher psychopathology in different domains, which in turn is related to worse therapy response, our second main hypothesis H2a is that higher PS at BL predicts more improvement in NSSI. Similarly, our secondary hypothesis H1b is that an increase in PS is associated with a reduction of BPD and depressive symptoms. Our secondary hypothesis H2b is that higher PS at BL predicts more improvement in BPD and depressive symptoms.

## Methods

### Participants

Patients with NSSI (incidents of NSSI on ⩾5 days in the last 12 months) were recruited from the specialized outpatient clinic for risk-taking and self-harm behavior (AtR!Sk; Ambulanz für Risikoverhalten & Selbstschädigung) at the Clinic for Child and Adolescent Psychiatry, University Hospital Heidelberg. At AtR!Sk, an open consultation hour, psychiatric diagnostic assessments and, if indicated, a subsequent treatment are offered. Treatment consisted of a cognitive-behavioral oriented short-term therapy (10 sessions), and a subsequent dialectic-behavioral therapy if required (Kaess, Ghinea, Fischer-Waldschmidt, & Resch, 2017). In addition, inpatient treatment was offered if indicated. Patients were also invited to participate in the nested AtR!Sk-Bio cohort, where neurobiological assessments were conducted. Inclusion criteria comprised: age 12–17 years, completed diagnostic and PS assessment at BL and annual FU, and informed and written consent of adolescents and their caregivers. Exclusion criteria comprised: acute psychotic symptoms, pregnancy, severe cardiovascular, neurological or endocrine diseases, and poor comprehension of German language. The Ethics Committee of the Faculty of Medicine, University of Heidelberg, approved the scientific evaluation of AtR!Sk (IRB approval number S-449/2013) and the add-on neurobiological assessments (IRB approval number S-514/2015).

### Procedure and measures

BL and yearly FU assessments (±2 months) took place at the Clinic for Child and Adolescent Psychiatry, University Hospital Heidelberg. For the current study, data at BL and 1-year FU were analyzed. The assessments at each time point were carried out at two separate appointments. The first appointment consisted of a sociodemographic anamnesis and several clinical diagnostic instruments. Relevant instruments for the current study are the *Self-Injurious Thoughts and Behaviors Interview* (SITBI-G) for the assessment of self-injurious thoughts and behaviors (Fischer et al., 2014), the *Structured Clinical Interview for DSM-IV Axis II Personality Disorders* (SCID-II) for the assessment of BPD symptoms (Wittchen, Zaudig, & Fydrich, 1997), and the *Depressionsinventar für Kinder und Jugendliche* (DIKJ) for a dimensional self-assessment of depressive symptoms (Stiensmeier-Pelster, Schürmann, & Duda, 2000). The second appointment took place max. 6 weeks after the first one, started at 8 a.m. and consisted of an anamnesis and several neurobiogenetic measurements, of which the measurement of PS is relevant for the current study. For the assessment of PS, an AHP-1800CPV Versatile Cold/Hot Plate (TECA Corp., Chicago, IL, USA) and a predefined programmed sequence for the temperature were used. Participants were instructed to place their non-dominant hand flat on the plate as soon as the BL temperature of 32 °C was reached. After a 3 min adaptation phase, temperature was increasing linearly by 1 °C across 13.3 s. Temperature at the first pain sensation (pain threshold) and temperature at intolerable pain sensation (pain tolerance) were measured in °C. As soon as pain was sensed, participants were instructed to continuously rate pain intensity on a digital visual analogue scale from 0 to 100 by indicating the value with a cursor with their dominant hand until pain tolerance was reached. If pain tolerance was not reached at 50 °C, the sequence ended automatically, and participants were asked to remove their hand to avoid tissue damage. We included several control variables in our analyses, since these variables have been associated with PS, namely age (Fillingim, 2005; Koenig et al., 2016), gender (Bartley & Fillingim, 2013; Dao & LeResche, 2000; Fillingim, 2005), medication (Sakhaie, Sadegzadeh, Dehghany, Adak, & Hakimeh, 2020), smoking (Al'Absi, Nakajima, & Grabowski, 2013; de Vita, Maisto, Ansell, Zale, & Ditre, 2019; Girdler et al., 2005), and coffee consumption (Overstreet, 2018). Smoking was found to be positively associated with PS in one study (de Vita et al., 2019) and negatively associated with PS in another (Girdler et al., 2005). Higher habitual caffeine consumption was found to be associated with lower PS (Overstreet, 2018).

### Statistical analysis

All analyses were performed using Stata (Version 17; StataCorp LP, College Station, TX, USA) with the significance level set to *α* = 0.05. Prior to analyses, PS parameters were standardized and change variables of the PS parameters were created by calculating the difference between scores at FU and at BL. Since nobody or only one person reported coffee consumption on the day of the experiment at BL or at FU, respectively, we did not include coffee consumption as a control variable in our analyses. Sociodemographic and clinical variables were tested for differences between BL and FU using two-sided pair-wise *t* tests for continuous variables, Wilcoxon sign-rank test for ordinal variables (i.e. NSSI frequency) and McNemar's χ^2^ test for categorical variables (i.e. smoking, medication). To explore the cross-sectional associations between our PS parameters as well as PS and psychopathology, we conducted Pearson product-moment correlations if both variables were continuous (pain threshold, pain tolerance, number of SCID-II BPD criteria, DIKJ, *Clinical Global Impressions-Severity*, and *Global Assessment of Functioning Scale*) and Spearman rank correlation if one variable was discrete (NSSI frequency). To test our hypotheses, we first calculated unadjusted models using pain threshold/tolerance and NSSI frequency/number of SCID-II BPD criteria/DIKJ symptoms as independent variables. We then adjusted models for our control variables (i.e. age, gender, medication, and smoking). To test H1a, we conducted a negative binomial regression with NSSI frequency in the last month at FU as dependent variable and change in pain threshold/tolerance and NSSI frequency in the last month at BL as independent variables. For the adjusted model, age at BL, gender (female = 1, male = 2), change in medication (medication at BL, but not at FU = 2, medication at FU, but not at BL = 1, no change = 0), and change in smoking during the last 12 months (increase = 2, decrease = 1, no change = 0) were added. Furthermore, as it is of clinical relevance to predict whether patients completely remitted regarding NSSI, a logistic regression analog to the negative binomial regression described above was conducted but with NSSI in the last month at FU as a dichotomous variable (yes = 1, no = 0). For the negative binomial and logistic regressions, the variable NSSI frequency in the last month at BL was added with a constant (+1) and then log transformed. This was done to bring it onto the same scale as the dependent variable NSSI frequency at FU and to make its regression coefficient interpretable. To test H1b, linear regressions were conducted with number of SCID-II BPD criteria or DIKJ symptoms, respectively, at FU as dependent variable and change in pain threshold/tolerance and number of SCID-II BPD criteria or DIKJ symptoms at BL, respectively, as independent variables. For the adjusted models, age at BL, gender, change in medication, and change in smoking during the last 12 months were added. To test H2, regression analyses analog to the ones for testing H1 were conducted but with pain threshold/tolerance at BL instead of change in pain threshold/tolerance as independent variable and medication (yes = 1, no = 0) and smoking (⩾once per week = 1, <once per week = 0) at BL instead of change in medication/smoking as control variables. To account for alpha inflation due to multiple testing of the same main hypotheses (H1a and H2a, respectively) with different statistical tests, i.e. negative binomial and logistic regression, we corrected the *p* values of the independent variables of interest (i.e. pain threshold and pain tolerance, respectively) in our unadjusted analyses using Holm, a sequentially rejective multiple test procedure, with *N* = 2 tests for each of the four subsets of our main hypotheses: (1) H1a with pain threshold as independent variable, (2) H1a with pain tolerance as independent variable, (3) H2a with pain threshold as independent variable, and (4) H2a with pain tolerance as independent variable (Holm, 1979). Regression coefficients were incident rate ratio (IRR) for negative binomial regression and odds ratio (OR) for logistic regression. In order to interpret our results with regard to the theories of both pain analgesia, and habituation, we conducted post-hoc linear regression analyses analog to the ones for H1a and H2a. In these analyses, independent variables were change in NSSI (instead of change in PS) and PS at BL (instead of NSSI at BL) (H1a) and the dependent variable was PS at FU (instead of NSSI at FU) (H1a and H2a).

## Results

### Sample characteristics and correlations between variables

The final sample size for analyses was *N* = 66, with *n* = 58 female and *n* = 8 male adolescents. At BL, participants reported to have injured themselves for around 2 years on average (*M* = 2.14, s.d. = 1.93). The most frequently reported methods of NSSI were cutting (98.45% at BL and FU), manipulating wounds (34.85% at BL and FU), hitting oneself (34.85% at BL, 33.33% at FU), and skin scratching (24.24% at BL, 37.88% at FU). Sociodemographic and clinical characteristics of the sample by time of assessment are provided in Table 1. At FU, participants showed fewer days with NSSI incidents (*z* = −4.73, *p* < 0.001) and less depressive symptoms (*t*[42] = −5.23, *p* < 0.001). The number of SCID-II BPD criteria did not significantly change between BL and FU (*t*[65] = −0.76, *p* = 0.452), neither did pain threshold (*t*[65] = −1.75, *p* = 0.084), pain tolerance (*t*[65] = −1.02, *p* = 0.313), pain intensity at pain tolerance (*t*[54] = 1.66, *p* = 0.102) nor medication intake (χ*^2^*[1] = 3.27, *p* = 0.071) change between assessments. At FU, more adolescents reported smoking at least once per week in the past 12 months compared to BL (χ^2^[1] = 11.84, *p* < 0.001). In total, 86.38% of the participants received therapy between BL and FU, with an average treatment duration of 33.5 sessions/days, including outpatient (on average 18.9 sessions) and inpatient/semi-residential treatment (on average 14.5 days). Cross-sectional correlation analyses of PS parameters showed significant but moderate correlations between pain threshold and pain tolerance (BL: *r* = 0.69, *p* < 0.001; FU: *r* = 0.44, *p* < 0.001). Cross-sectional correlation analyses of PS parameters with measures of psychopathology revealed only one significant correlation between DIKJ and pain threshold at FU (*r* = 0.34, *p* = 0.02).

**Table 1.** Sociodemographic and clinical characteristics of the study sample by time

Variable	Baseline (*N* = 66)	Follow-up (*N* = 66)	Effect size^a^	Test statistic, *p* value^b^
Age (yr)	15.24 (1.43)	16.27 (1.43)	*d* = 0.72	*t*(65) = 23.96, *p* < 0.001
Weight (kg)	60.32 (15.67)	62.21 (15.58)	*d* = 0.12	*t*(65) = 4.25, *p* < 0.001
Height (cm)	166.89 (7.80)	167.92 (7.67)	*d* = 0.13	*t*(65) = 3.89, *p* < 0.001
BMI	21.49 (4.45)	21.92 (4.42)	*d* = 0.10	*t*(65) = 2.55, *p* = 0.013
DIKJ^c^	30.75 (9.49)	21.12 (12.36)	*d* = −0.86	*t*(42) = −5.23, *p* < 0.001
Number of SCID-II BPD criteria	3.15 (2.08)	2.95 (2.23)	*d* = −0.12	*t*(65) = −0.76, *p* = 0.452
Number of NSSI episodes in the past month	6.83 (8.12)	2.11 (5.13)	*r* = - 0.58	*z* = −4.73, *p* < 0.001
Pain threshold	42.49 (3.87)	41.73 (3.08)	*d* = −0.21	*t*(65) = −1.75, *p* = 0.084
Pain tolerance	47.16 (2.12)	46.89 (2.20)	*d* = −0.12	*t*(65) = −1.02, *p* = 0.313
Pain intensity at pain tolerance	81.24 (2.73)	86.81 (2.28)	*d* *=* 0.30	*t*(54) = 1.66, *p* = 0.102
Smoking (⩾once per week in the past 12 months, in %)	24.24	46.97	*d* = 0.94	χ^2^(1) = 11.84, *p* < 0.001
Medication intake (yes, in %)	19.70	30.30	*d* = 0.46	χ^2^(1) = 3.27, *p* = 0.071
Caffeine consumption (yes, in %)	0	0.02	*d* = 0.25	χ^2^(1) = 1.00, *p* = 0.317

*Notes.* All values are means and standard deviations (s.d.) in brackets if not otherwise indicated; BMI, body mass index; BPD, borderline personality disorder; DIKJ, *Depressionsinventar für Kinder und Jugendliche*; NSSI, non-suicidal self-injury; SCID-II, Structured Clinical Interview for DSM-IV Axis II Personality Disorders.

a Small effect: |*d*|  ⩾ 0.2; |*r*| ⩾ 0.1. Medium effect: |*d*|  ⩾ 0.5; |*r*|  ⩾ 0.3. Large effect: *d*  ⩾ 0.8; |*r*|  ⩾ 0.5. Effect sizes for categorical variables were calculated from χ^2 35^.

b Values refer to differences between baseline and follow-up, with *t* tests for continuous variables, Wilcoxon signed-rank test for ordinal variables and McNemar's χ^2^ test for categorical variables.

c Due to missing data *N* for DIKJ was 43 with *n* = 37 female adolescents and *N* for pain intensity was 55 with *n* = 48 female adolescents.

### Longitudinal covariance of PS with NSSI frequency, BPD, and depression

**Figure 1.**
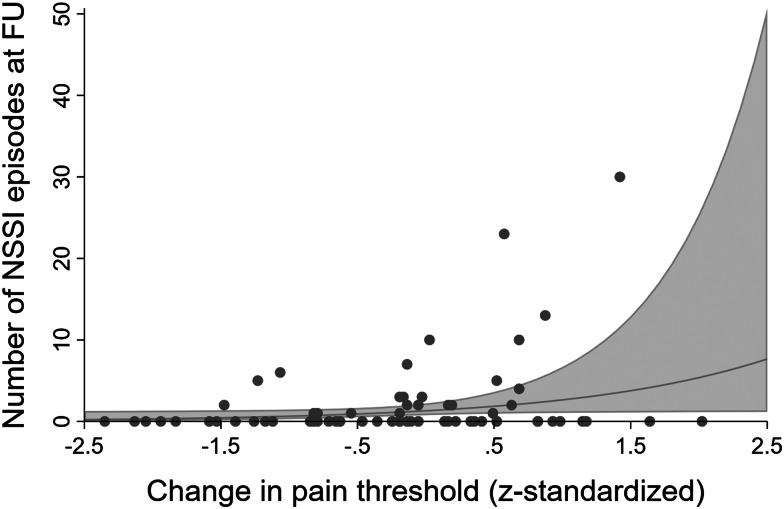
Predicted number of non-suicidal self-injury episodes at follow-up by change in pain threshold after controlling for age, gender, medication, and smoking. FU, follow-up; NSSI, non-suicidal self-injury.

Change in pain threshold predicted NSSI in the last month at FU in the unadjusted (IRR = 2.23, *p* = 0.012) as well as in the adjusted model (IRR = 2.04, *p* = 0.047, see Fig. 1): an increase in pain threshold by one standard deviation predicted around twice the number of days with NSSI in the last month at FU. This effect was still significant after Holm correction (*p* = 0.025). When treating NSSI as a dichotomous variable, this association was not found: change in pain threshold did not predict whether participants did or did not injure themselves in the last month at FU (unadjusted OR = 1.29, *p* = 0.379; adjusted OR = 1.28, *p* = 0.416) (detailed results are reported in online Supplement A). Change in pain tolerance did not predict NSSI frequency in the last month at FU, neither in the negative binomial regression (unadjusted IRR = 1.41, *p* = 0.434; adjusted IRR = 2.16, *p* = 0.065) nor in the logistic regression (unadjusted OR = 1.31, *p* = 0.416; adjusted OR = 1.56, *p* = 0.225) although results were close to significant in the adjusted negative binomial regression. Further analyses revealed no significant associations between any of the PS parameters (change in pain threshold or tolerance) with BPD (pain threshold: *b* = 0.37, *p* = 0.143; pain tolerance: *b* = 0.17, *p* = 0.555 for unadjusted models and pain threshold: *b* = 0.40, *p* = 0.136; pain tolerance: *b* = 0.24, *p* = 0.451 for adjusted models) or depression (pain threshold: *b* = 0.97, *p* = 0.569; pain tolerance: *b* = 1.71, *p* = 0.489 for unadjusted models and pain threshold: *b* = 0.84, *p* = 0.650; pain tolerance: *b* = 1.96, *p* = 0.461 for adjusted models), respectively. Post-hoc analyses with change in NSSI frequency and pain threshold/pain tolerance at BL as independent variables and pain threshold/pain tolerance at FU as dependent variable did not yield any significant effects (for detailed results see online Supplement C). Change in NSSI frequency neither predicted pain threshold (unadjusted *b* = 0.01, *p* = 0.527; adjusted *b* < 0.01, *p* = 0.678) nor pain tolerance (unadjusted *b* = 0.01, *p* = 0.508; adjusted *b* = 0.01, *p* = 0.292) at FU.

### PS as a predictor for change in NSSI frequency, BPD, and depression

**Figure 2.**
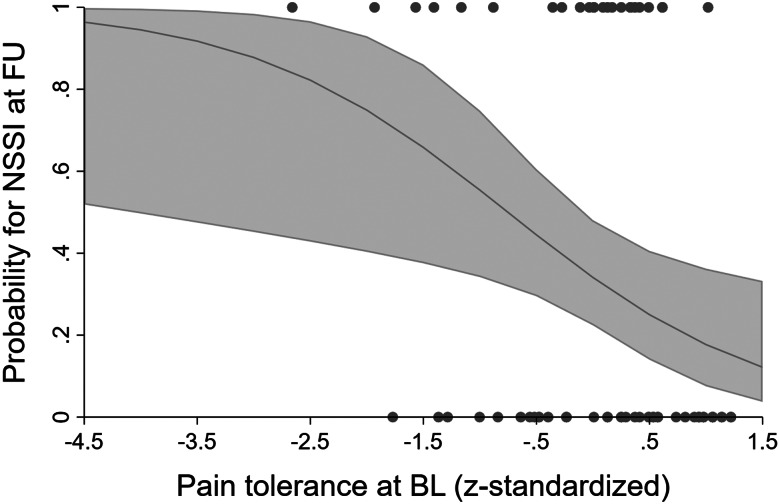
Predicted probabilities for non-suicidal self-injury at follow-up by pain tolerance at baseline after controlling for age, gender, medication, and smoking. BL, baseline; FU, follow-up; NSSI, non-suicidal self-injury.

Detailed results of these analyses are reported in online Supplement B. Pain threshold at BL did not predict NSSI frequency in the last month at FU neither in the negative binomial regression (unadjusted IRR = 0.85, *p* = 0.654; adjusted IRR = 0.68, *p* = 0.296) nor in the logistic regression (unadjusted OR = 0.83, *p* = 0.465; adjusted OR = 0.84, *p* = 0.516). Pain tolerance at BL did not predict NSSI frequency in the last month at FU in the negative binomial regression (unadjusted IRR = 0.69, *p* = 0.340; adjusted IRR = 0.50, *p* = 0.074), but did predict whether participants did or did not injure themselves in the last month at FU in the logistic regression in the unadjusted (OR = 0.49, *p* = 0.030) as well as in the adjusted (OR = 0.42, *p* = 0.016, see Fig. 2) model. Higher pain tolerance at BL predicted lower probability for NSSI episodes in the last month at FU. However, after Holm correction, this effect was not significant anymore (*p* *=* 0.059). Further analyses revealed no significant associations between any of the PS parameters (pain threshold or tolerance at BL) with BPD (pain threshold: *b* = −0.13, *p* = 0.580; pain tolerance: *b* = −0.11, *p* = 0.692 for unadjusted models and pain threshold: *b* = −0.11, *p* = 0.643; pain tolerance: *b* = −0.10, *p* = 0.740 for adjusted models) or depression (pain threshold: *b* = 1.78, *p* = 0.275; pain tolerance: *b* = 2.54, *p* = 0.228 for unadjusted models and pain threshold: *b* = 1.02, *p* = 0.537; pain tolerance: *b* = 1.04, *p* = 0.634 for adjusted models), respectively. Post-hoc analyses with pain threshold/pain tolerance instead of NSSI at FU as dependent variable did not yield any significant effects. NSSI frequency at BL neither predicted pain threshold (unadjusted/adjusted *b* = 0.01, *p* = 0.385) nor pain tolerance (unadjusted *b* = −0.01, *p* = 0.476; adjusted *b* = −0.01, *p* = 0.556) at FU.

## Discussion

The present study sought to investigate (1) the longitudinal covariance of PS with NSSI frequency, BPD, and depressive symptoms and (2) whether PS at BL predicts change in NSSI frequency, BPD, and depressive symptoms in adolescents with NSSI over 1 year. Results partly confirmed our first main hypothesis. We found a decrease in pain threshold to be associated with improvement in NSSI (H1a). This is in line with the cross-sectional study from Ludäscher et al. (2009) that suggests that pain threshold may normalize following cessation of NSSI. Hence, pain threshold seems to reflect a state rather than a trait in NSSI. Since a decrease in NSSI frequency did not predict change in PS, our results suggest that the decrease in pain threshold cannot simply be explained by sensitization to pain due to reduced engagement in NSSI. Other mechanisms, yet to explore, have led to a decrease in pain threshold which in turn may lead to a decrease in NSSI frequency. Therefore, our results support the pain analgesia hypothesis which states PS as a risk factor for NSSI.

In line with Koenig et al. (2017a), we did not find an association between change in PS and change in BPD or depressive symptoms after 1 year. Compared with cross-sectional studies, our study is also consistent with the study from Ludäscher et al. (2009) which did not find an association between BPD symptoms and pain threshold. In contrast, Van der Venne et al. (2021) found an association between pain threshold and the number of BPD symptoms, using the same pain stimulation paradigm and a sample that partly overlaps with the sample reported on in this study. However, cross-sectional studies do not allow for investigating the relationship between change in these variables and can thus only partly be compared to our study. Cross-sectional studies that showed an association between depressive symptoms and PS either investigated adults, measured PS differently, and modeled depression as a categorical diagnosis instead of a continuum (Kim et al., 2022; Thompson et al., 2016) or found another parameter of PS, i.e. pain intensity, to be associated with depressive symptoms (Van der Venne et al., 2021).

Contrary to our hypotheses, lower pain threshold at BL did not predict more improvement in clinical symptoms (NSSI frequency, BPD, and depressive symptoms). This is in contrast to the idea that lower PS reflects higher psychopathology and should therefore predict less improvement in clinical symptoms. In line with this, several studies did not find an association between pain threshold and tolerance with overall psychopathology (Glenn et al., 2014; Ludäscher et al., 2015) or severity of psychiatric symptoms (Van der Venne et al., 2021). Our analyses of correlations between PS parameters and measures of psychopathology only revealed one significant correlation between DIKJ and pain threshold at FU (*r* = 0.34, *p* = 0.02) and the directions of correlations were inconsistent across different measures of psychopathology or even PS. Taken together, this suggests that the association between PS and psychopathology is complex.

While lower pain *threshold* at BL did not predict more improvement in clinical symptoms, we found that higher pain *tolerance* at BL predicted lower probability for NSSI episodes in the last month at FU. Hence, higher pain tolerance seems to predict potential remission of NSSI, which would argue against the pain analgesia hypothesis. This might seem counterintuitive at first and must be interpreted with caution, since the effect was not significant anymore after Holm correction. Nevertheless, we will provide some possible explanations for this finding. Pain threshold and pain tolerance measure different aspects of PS and should therefore be interpreted differently. Our analyses revealed significant but moderate correlations between pain threshold and pain tolerance, suggesting PS being a rather heterogeneous than a homogeneous construct. As such, it is possible that an individual displays a low pain threshold but high pain tolerance. In line with this, Schmitz, Vierhaus, and Lohaus (2013) found that the endurance of pain is not influenced by one's pain threshold. Pain threshold might be more dependent on physiological factors and relatively stable within a person, whereas pain tolerance is strongly modulated by psychological factors, like self-efficacy and coping (Schmitz et al., 2013; Tesarz, Schuster, Hartmann, Gerhardt, & Eich, 2012). Consistently, Feldner and Hekmat (2001) showed that perceived control over anxiety-related events influences pain tolerance, but not pain threshold. This can be interpreted as follows: perceiving control over an anxiety-inducing situation affects one's response to pain, which is reflected by pain tolerance and pain endurance, but does not affect the subjective experience of pain, which is reflected by, e.g. pain threshold. Furthermore, our results suggest that pain threshold and tolerance are differently associated with NSSI: we found a decrease in pain threshold to predict reduced NSSI frequency at FU which suggests a positive relationship between pain threshold and NSSI, whereas pain tolerance seems to display a negative relationship with NSSI. This is consistent with the finding that an increase in pain tolerance is associated with a reduction in NSSI frequency (Koenig et al., 2017a). A possible explanation for why higher pain tolerance might be protective against NSSI is that the pain stimulus is no longer perceived as aversive enough to effectively relieve stress. However, a recent longitudinal study with young adults engaging in NSSI showed that higher pain threshold and tolerance at BL predicted higher NSSI versatility, i.e. number of different NSSI methods, in the past year at 1 year FU (Boyne & Hamza, 2022). Higher pain threshold at BL also predicted higher NSSI frequency in the past year, even though this result was not significant anymore after Bonferroni correction. Hence, in contrast to our study, this suggests that higher pain threshold and tolerance may be a risk factor for NSSI. A reason for discrepant findings between this study and ours might be differences in sample characteristics. While their sample consisted of students, ours consisted of patients. Therefore, the severity of NSSI and level of PS might differ between these samples. It could be hypothesized that only within a certain window of PS, NSSI is considered an effective method to relieve stress: if PS is too high, one might not engage in NSSI since it is perceived as too painful; if PS is too low, NSSI might not be painful enough to relieve stress. Assuming that a clinical sample displays a higher pain tolerance at BL than a non-clinical sample, this hypothesis might explain why lower pain tolerance in patients, but higher pain tolerance in students are both associated with higher risk for NSSI, since both levels of pain tolerance might fall into the window of PS where NSSI is considered effective. The results from our post-hoc analyses suggest that higher pain tolerance did not result from an increase in NSSI engagement. If patients had high pain tolerance throughout, this leaves the question open why these patients engaged at all in NSSI if it does not effectively relieve stress. There are two possible explanations. Like Boyne and Hamza (2022) suggested, pain habituation might be more relevant to NSSI onset *v.* maintenance. Since our sample was injuring themselves for more than 2 years already, continued NSSI might not reduce pain tolerance anymore, but NSSI at onset might have reduced pain tolerance. Longitudinal studies that follow adolescents who are at risk for NSSI, but do not display this behavior yet or only rarely/less severely over several years would be necessary to investigate this. Another possibility is that in some patients pain tolerance is already high at NSSI onset. Emotion regulation through painful experience (Doukas et al., 2020) might not be the main function of NSSI for these patients since the self-injury might not induce a strong physical sensation. Instead, interpersonal functions, such as increasing attention and support (Nock, 2010), or ending depersonalization or numbness by seeing blood (Bresin & Gordon, 2013) might be more relevant. A potential explanation for why these patients may have a better prognosis regarding remission might be that problems other than emotion regulation can be treated more effectively.

As pain tolerance seems to be malleable by psychological factors and higher pain tolerance might be protective against NSSI, increasing pain tolerance by modifying cognitions or coping with regard to pain might be an approach to indirectly target NSSI in psychotherapy for adolescents who fall into the suggested risk window of PS. There is evidence for mindfulness-based interventions to reduce PS (Andersen et al., 2021; Ferreira-Valente et al., 2022; Wilson, Haliwa, Lee, & Shook, 2023). However, as we do not know how to determine the risk and target pain tolerance yet, it is not possible to plan such interventions. Further, increasing pain tolerance might confer risks like trying more severe NSSI methods. Another idea would be to reduce pain tolerance to the point people feel averse to injure themselves. Opioid antagonists like naltrexone can successfully treat NSSI, possibly by reducing pain tolerance (Bandelow & Wedekind, 2015; Bresin & Gordon, 2013). However, reduced pain tolerance might also confer risks like being more prone to pain disorders (McDermid, Rollman, & McCain, 1996).

In our study, no associations between PS and BPD or depression were observed. Since only NSSI was associated with PS, our study might suggest that altered PS is a unique marker for NSSI and rather independent from its underlying psychopathology. This is supported by Koenig et al. (2017a) who found only changes in NSSI frequency and pain tolerance to be correlated, whereas no such correlations were found for depression or BPD. Also, Ludäscher et al. (2009) found that patients with BPD who currently injure themselves demonstrated lower PS than patients with BPD who have stopped injuring themselves. However, Koenig et al. (2016) found greater pain threshold in BPD compared to NSSI. Since the last two mentioned studies differed in methodology (e.g. adult or only female participants, cross-sectional design) from our study, they are difficult to compare. Future studies comparing individuals engaging in NSSI with different clinical etiology as well as studies comparing adolescents with and without a depressive disorder are necessary to clarify if or in which regard altered PS is a unique marker for NSSI. For example, it is conceivable that pain threshold might be a state marker for some clinical symptoms (e.g. NSSI) and a trait marker for others.

### Limitations and strengths

One of the limitations of our study concerns our pain stimulation paradigm which is an experimental operationalization for actual NSSI. Thus, PS in the context of NSSI might differ from PS in the context of thermal pain stimulation. However, the meta-analysis by Koenig et al. (2016) did not reveal a significant effect for modality of pain stimulation on PS. Still, it might be worthwhile for future studies to use pain stimuli more similar to those used by adolescents engaging in NSSI, like blunted blade stimuli which elicit similar patterns of affective and sensory pain experience as real incision (Shabes et al., 2016). Also, the validity of the pain tolerance measurement is constrained, since its maximum was artificially set to 50 °C. It is likely that a considerable number of participants would have tolerated a higher temperature than 50 °C since the distribution of pain tolerance is left-skewed. Another limitation is that we only assessed NSSI frequency, but not other indicators of NSSI severity, like NSSI versatility, which has been associated with PS (Boyne & Hamza, 2022). Taking into account both NSSI frequency and versatility might yield important insight in the future (Ammerman, Jacobucci, Turner, Dixon-Gordon, & McCloskey, 2020). To consider the effect of gender, we included it as a control variable and conducted sensitivity analyses. In none of the analyses gender displayed a significant main effect. Sensitivity analyses without males yielded similar results but change in pain threshold did not predict NSSI frequency in the last month anymore. However, due to the small number of male participants we are unable to conclude, if the relationship between change in pain threshold and NSSI is indeed different across gender. Exploratory visualization of the data did not suggest that the association between change in pain threshold and change in NSSI differs by gender. Another limitation of our study is that we did not account for the potential influence of ethnicity and sex hormones. Sex hormones were found to have a substantial impact on pain perception (Paller, Campbell, Edwards, & Dobs, 2009) and white people tend do display lower PS compared to Hispanics and African Americans (Fillingim, 2005; Grewen, Light, Mechlin, & Girdler, 2008; Rahim-Williams, Riley, Williams, & Fillingim, 2012). Also, even though we accounted for the influence of coffee consumption, we did not consider other substances containing caffeine. In addition to that, it remains unclear which factors contributed to the overall improvement in clinical symptoms. Due to the naturalistic design of our study, we were unable to control for unspecific effects outside of therapy. Further, even though the majority of participants received therapy, a small percentage did not. Therefore, we do not know if improvement in clinical symptoms can be interpreted as therapy response. Studies with randomized-controlled designs using clinical control groups (e.g. waitlist control group) are needed to determine whether pain tolerance can serve as a predictor for therapy response. Lastly, changes in PS might not evolve within 1 year (Koenig et al., 2017a). Thus, studies using repeated measures over longer time-periods might shed more light on the relationship between PS and NSSI development.

The strength of our study is the relatively large sample of well-characterized adolescents engaging in NSSI as well as the longitudinal design.

## Conclusion

To summarize, the present study found that (1) a decrease in pain threshold predicted reduced NSSI and (2) a higher pain tolerance at BL predicted lower probability for NSSI 1 year later, even though this latter finding did not survive Holm correction. This suggests, firstly, that pain threshold might normalize with decreasing frequency of NSSI and could thus serve as a state marker for NSSI. Secondly, it suggests that pain threshold and pain tolerance reflect different aspects of PS that seem to be differently associated with NSSI. Future studies comparing adolescents engaging in NSSI with different clinical etiologies are necessary to clarify if or in which regard altered PS is a unique marker for NSSI.

## Supporting information

Kao et al. supplementary material Kao et al. supplementary material

## Supplementary material

The supplementary material for this article can be found at https://doi.org/10.1017/S0033291724000461
