# Supplementary material for: Pain sensitivity as a state marker and predictor for adolescent non-suicidal self-injury
Source: Psychol Med. 2024 Mar 11;54(9):2291–8. doi: 10.1017/S0033291724000461 (PMC11413347; doi:10.1017/S0033291724000461)
Supplement: Kao et al. supplementary material [file S0033291724000461sup001.docx]

**Supplement A**

H1 Analyses: Longitudinal covariance of pain sensitivity with NSSI frequency, BPD and depression

|  |  | **Variable** | | | | | |
| --- | --- | --- | --- | --- | --- | --- | --- |
|  |  | NSSI^1^/ BPD/DIKJ | Pain threshold/ tolerance | Age | Gender | Medication^2^ | Smoking^2^ |
| Model 1 (*N* = 66):  DV = NSSI frequency,  IV = pain threshold | *IRR* (*SE*) | 1.92 (0.46);  *1.59 (0.35)* | 2.04 (0.73);  *2.23 (0.71)* | 1.41 (0.31) | 0.56 (0.53) | 0.32 (0.26) | 1.55 (0.95) |
|  | 95% CI | [1.21, 3.06];  *[1.04, 2.45]* | [1.01, 4.10];  *[1.19, 4.17]* | [0.92, 2.17] | [0.09, 3.54] | [0.07, 1.57] | [0.47, 5.13] |
|  | *z* | 2.76; *2.14* | 1.99; *2.50* | 1.59 | -0.61 | -1.40 | 0.72 |
|  | *p* | .006; *.032* | .047; *.012/.025*^3^ | .112 | .539 | .161 | .469 |
| Model 2 (*N* = 66):  DV = NSSI (yes/no),  IV = pain threshold | *OR* (*SE*) | 1.54 (0.39);  *1.52 (0.37)* | 1.28 (0.39);  *1.29 (0.38)* | 1.24 (0.27) | 0.49 (0.46) | 0.68 (0.53) | 0.97 (0.63) |
|  | 95% CI | [0.93, 2.53];  *[0.94, 2.45]* | [0.71, 2.33];  *[0.73, 2.28]* | [0.82, 1.89] | [0.07, 3.16] | [0.15, 3.16] | [0.27, 3.50] |
|  | *z* | 1.68; *1.70* | 0.81; *0.88* | 1.02 | -0.75 | -0.50 | -0.05 |
|  | *p* | .092; *.089* | .416; *.379/ .379*^3^ | .307 | .451 | .620 | .961 |
| Model 3 (*N* = 66):  DV = NSSI frequency,  IV = pain tolerance | *IRR* (*SE*) | 2.48 (0.65);  *1.79 (0.40)* | 2.16 (0.90);  *1.41 (0.62)* | 1.73 (0.40) | 0.71 (0.65) | 0.18 (0.16) | 2.03 (1.29) |
|  | 95% CI | [1.49, 4.14];  *[1.16, 2.78]* | [0.95, 4.87];  *[0.59, 3.35]* | [1.09, 2.73] | [0.12, 4.27] | [0.03, 1.02] | [0.59, 7.05] |
|  | *z* | 3.48; *2.60* | 1.85; *0.78* | 2.34 | -0.37 | -1.94 | 1.12 |
|  | *p* | <.001; *.009* | .065; *.434/ .434*^3^ | .019 | .712 | .052 | .265 |
| **Supplement A (continued)** | | | | | | | |
|  |  | **Variable** | | | | | |
|  |  | NSSI^1^/ BPD/DIKJ | Pain threshold/ tolerance | Age | Gender | Medication^2^ | Smoking^2^ |
| Model 4 (*N* = 66):  DV = NSSI (yes/no),  IV = pain tolerance | *OR* (*SE*) | 1.68 (0.44);  *1.61 (0.40)* | 1.56 (0.57);  *1.31 (0.44)* | 1.34 (0.30) | 0.50 (0.48) | 0.56 (0.46) | 1.02 (0.68) |
|  | 95% CI | [1.00, 2.80];  *[0.99, 2.61]* | [0.76, 3.18];  *[0.68, 2.52]* | [0.87, 2.07] | [0.08, 3.26] | [0.11, 2.83] | [0.28, 3.75] |
|  | *z* | 1.98; *1.93* | 1.21; *0.81* | 1.33 | -0.72 | -0.70 | 0.04 |
|  | *p* | .048; *.054* | .225; *.416/ .832*^3^ | .185 | .469 | .484 | .972 |
| Model 5 (*N* = 66):  DV = BPD,  IV = pain threshold | *b* (*SE*) | 0.57 (0.12);  *0.56 (0.11)* | 0.40 (0.26);  *0.37 (0.25)* | -0.07 (0.19) | 0.23 (0.76) | -0.67 (0.68) | -0.24 (0.58) |
|  | 95% CI | [0.33, 0.81];  *[0.33, 0.78]* | [-0.13, 0.92];  *[-0.13, 0.86]* | [-0.45, 0.31] | [-1.30, 1.76] | [-2.04, 0.70] | [-1.41, 0.92] |
|  | *t* | 4.70; *4.92* | 1.51; *1.48* | -0.37 | 0.30 | -0.97 | -0.42 |
|  | *p* | <.001, *<.001* | .136, *.143* | .713 | .764 | .334 | .676 |
| Model 6 (*N* = 66):  DV = BPD,  IV = pain tolerance | *b* (*SE*) | 0.54 (0.13);  *0.55 (0.12)* | 0.24 (0.31);  *0.17 (0.28)* | 0.00 (0.20) | 0.29 (0.77) | -0.65 (0.71) | -0.17 (0.59) |
|  | 95% CI | [0.29, 0.79];  *[0.31, 0.78]* | [-0.39, 0.86];  *[-0.40, 0.73]* | [-0.39, 0.40] | [-1.25, 1.84] | [-2.10, 0.76] | [-1.36, 1.02] |
|  | *t* | 4.28; *4.66* | 0.76; *0.59* | 0.02 | 0.38 | -0.92 | -0.29 |
|  | *p* | <.001; *<.001* | .451, *.555* | .984 | .706 | .359 | .774 |
| **Supplement A (continued)** | | | | | | | |
|  |  | **Variable** | | | | | |
|  |  | NSSI^1^/ BPD/DIKJ | Pain threshold/ tolerance | Age | Gender | Medication^2^ | Smoking^2^ |
| Model 7 (*N* = 43):  DV = DIKJ,  IV = pain threshold | *b* (*SE*) | 0.47 (0.20);  *0.52 (0.19)* | 0.84 (1.84);  *0.97 (1.68)* | 0.10 (2.03) | 6.01 (5.91) | -1.24 (6.85) | 3.69 (4.65) |
|  | 95% CI | [0.07, 0.88];  *[0.14, 0.90]* | [-2.90, 4.59];  *[-2.44, 4.37]* | [-4.02, 4.22] | [-6.00, 18.01] | [-15.16, 12.69] | [-5.77, 13.14] |
|  | *t* | 2.39; *2.78* | 0.46; *0.57* | 0.05 | 1.02 | -0.18 | 0.79 |
|  | *p* | .023, *.008* | .650, *.569* | .960 | .316 | .858 | .434 |
| Model 8 (*N* = 43):  DV = DIKJ,  IV = pain tolerance | *b* (*SE*) | 0.45 (0.20);  *0.51 (0.19)* | 1.96 (2.62);  *1.71 (2.45)* | 0.35 (1.95) | 6.05 (5.87) | -1.95 (6.92) | 4.45 (4.68) |
|  | 95% CI | [0.04, 0.86];  *[0.13, 0.90]* | [-3.38, 7.29];  *[-3.24, 6.66]* | [-3.60, 4.31] | [-5.89, 17.99] | [-16.02, 12.12] | [-5.07, 13.96] |
|  | *t* | 2.25; *2.69* | 0.75; *0.70* | 0.18 | 1.03 | -0.28 | 0.95 |
|  | *p* | .031; *.010* | .461; *.489* | .856 | .311 | .780 | .349 |

*Notes.* All dependent variables are measured at follow-up. The independent variables NSSI, BPD, DIKJ, age and gender are measured at baseline. The independent variables pain threshold, pain tolerance, medication and smoking are change variables (difference between follow-up and baseline). Values in italics refer to unadjusted analyses without control variables. BPD = Borderline personality disorder; DIKJ = *Depressionsinventar für Kinder und Jugendliche*; DV = dependent variable; IV = independent variable; NSSI = non-suicidal self-injury.

^1^ log-transformed.

^2^ Level 1 compared to level 0.

^3^ After correction for multiple testing using Holm.

**Supplement B**

H2 Analyses: Pain sensitivity at baseline as predictor for change in NSSI frequency, BPD and depression

|  |  | Variable | | | | | |
| --- | --- | --- | --- | --- | --- | --- | --- |
|  |  | NSSI^1^/ BPD /DIKJ | Pain threshold/ tolerance | Age | Gender | Medication | Smoking |
| Model 1 (*N* = 66):  DV = NSSI frequency,  IV = pain threshold | *IRR* (*SE*) | 1.78 (0.45);  *1.70 (0.40)* | 0.68 (0.25);  *0.85 (0.31)* | 1.45 (0.31) | 2.15 (2.50) | 0.66 (0.67) | 1.71 (1.20) |
|  | 95% CI | [1.09, 2.91];  *[1.08, 2.68]* | [0.32, 1.41];  *[0.42, 1.73]* | [0.95, 2.21] | [0.22, 20.87] | [0.09, 4.80] | [0.43, 6.75] |
|  | *z* | 2.30; *2.28* | -1.04; *-0.45* | 1.70 | 0.66 | -0.41 | 0.77 |
|  | *p* | .022; *.022* | .296; *.654/.654*^2^ | .089 | .509 | .680 | .442 |
| Model 2 (*N* = 66):  DV = NSSI (yes/no), IV = pain threshold | *OR* (*SE*) | 1.70 (0.46);  *1.52 (0.37)* | 0.84 (0.22);  *0.83 (0.21)* | 1.21 (0.25) | 0.71 (0.66) | 0.57 (0.42) | 2.85 (1.88) |
|  | 95% CI | [1.00, 2.91];  *[0.94, 2.46]* | [0.50, 1.41];  *[0.51, 1.36]* | [0.81, 1.81] | [0.11, 4.42] | [0.14, 2.44] | [0.78, 10.41] |
|  | *z* | 1.95; *1.72* | -0.65; *-0.73* | 0.92 | -0.37 | -0.75 | 1.59 |
|  | *p* | .051; *.086* | .516; *.465/.931*^2^ | .360 | .715 | .452 | .113 |
| Model 3 (*N* = 66):  DV= NSSI frequency,  IV = pain tolerance | *IRR* (*SE*) | 1.90 (0.47);  *1.73 (0.38)* | 0.50 (0.19);  *0.69 (0.27)* | 1.55 (0.33) | 2.06 (2.22) | 0.80 (0.75) | 1.56 (1.01) |
|  | 95% CI | [1.17, 3.10];  *[1.13, 2.65]* | [0.24, 1.07];  *[0.32, 1.49]* | [1.02, 2.36] | [0.25, 16.94] | [0.13, 5.06] | [0.44, 5.53] |
|  | *z* | 2.57; *2.50* | -1.79; *-0.95* | 2.06 | 0.67 | -0.24 | 0.69 |
|  | *p* | .010; *.012* | .074; *.340/.340*^2^ | .039 | .501 | .812 | .490 |
| **Supplement B (continued)** | | | | | | | |
|  |  | **Variable** | | | | | |
|  |  | NSSI^1^/ BPD /DIKJ | Pain threshold/ tolerance | Age | Gender | Medication | Smoking |
| Model 4 (*N* = 66):  DV= NSSI (yes/no), IV = pain tolerance | *OR* (*SE*) | 1.92 (0.56);  *1.64 (0.42)* | 0.42 (0.15);  *0.49 (0.16)* | 1.37 (0.31) | 0.97 (0.94); | 0.57 (0.43) | 3.18 (2.23) |
|  | 95% CI | [1.08, 3.42];  *[0.99, 2.72]* | [0.20, 0.85];  *[0.26, 0.93]* | [0.88, 2.14] | [0.15, 6.47] | [0.13, 2.54] | [0.81, 12.54] |
|  | *z* | 2.23; *1.93* | -2.40; *-2.18* | 1.40 | -0.03 | -0.74 | 1.65 |
|  | *p* | .026; .*054* | .016, *.030/.059*^2^ | .162 | .975 | .458 | .099 |
| Model 5 (*N* = 66):  DV = BPD,  IV = pain threshold | *b* (*SE*) | 0.52 (0.13);  *0.57 (0.12)* | -0.11 (0.23);  *-0.13 (0.23)* | -0.02 (0.18) | 0.40 (0.75) | -0.50 (0.64) | 0.85 (0.61) |
|  | 95% CI | [0.26, 0.78];  *[0.34, 0.80]* | [-0.57, 0.36];  *[-0.59, 0.33]* | [-0.39, 0.34] | [-1.11, 1.90] | [-1.78, 0.78] | [-0.36, 2.06] |
|  | *t* | 3.97; *4.87* | -0.47; *-0.56* | -0.13 | 0.53 | -0.79 | 1.40 |
|  | *p* | <.001; *<.001* | .643; *.580* | .899 | .601 | .434 | .165 |
| Model 6 (*N* = 66):  DV = BPD,  IV = pain tolerance | *b* (*SE*) | 0.51 (0.13);  *0.56 (0.12)* | -0.10 (0.30);  *-0.11 (0.28)* | -0.01 (0.18) | 0.42 (0.76) | -0.52 (0.64) | 0.85 (0.61) |
|  | 95% CI | [0.25, 0.77];  *[0.33, 0.80]* | [-0.68, 0.49];  *[-0.68; 0.45]* | [-0.38, 0.36] | [-1.10, 1.94] | [-1.80, 0.75] | [-0.37, 2.06] |
|  | *t* | 3.96; *4.85* | -0.33; *-0.40* | -0.06 | 0.55 | -0.82 | 1.39 |
|  | *p* | <.001, *<.001* | .740; *.692* | .952 | .582 | .414 | .169 |
| **Supplement B (continued)** | | | | | | | |
|  |  | **Variable** | | | | | |
|  |  | NSSI^1^/ BPD /DIKJ | Pain threshold/ tolerance | Age | Gender | Medication | Smoking |
| Model 7 (*N* = 43):  DV = DIKJ,  IV = pain threshold | *b* (*SE*) | 0.62 (0.18);  *0.55 (0.19)* | 1.02 (1.64);  *1.78 (1.61)* | -0.79 (1.78) | 5.86 (5.15) | 4.79 (4.80) | 10.32 (4.65) |
|  | 95% CI | [0.25, 0.99];  *[0.18, 0.93]* | [-2.30, 4.34];  *[-1.47, 5.04]* | [-4.41, 2.83] | [-4.59, 16.31] | [-4.95, 14.53] | [0.89, 19.74] |
|  | *t* | 3.42; *2.98* | 0.62; *1.11* | -0.44 | 1.14 | 1.00 | 2.22 |
|  | *p* | .002; *.005* | .537; *.275* | .660 | .263 | .326 | .033 |
| Model 8 (*N* = 43):  DV = DIKJ,  IV = pain tolerance | *b* (*SE*) | 0.63 (0.18);  *0.56 (0.18)* | 1.04 (2.16);  *2.54 (2.08)* | -0.91 (1.76) | 5.54 (5.27) | 4.55 (4.93) | 10.50 (4.65) |
|  | 95% CI | [0.25, 1.00];  *[0.19, 0.93]* | [-3.34, 5.41];  *[-1.65, 6.74]* | [-4.48, 2.66] | [-5.15, 16.23] | [-5.46, 14.56] | [1.07, 19.92] |
|  | *t* | 3.42; *3.03* | 0.48; *1.23* | -0.52 | 1.05 | 0.92 | 2.26 |
|  | *p* | .002; *.004* | .634; *.228* | .608 | .300 | .363 | .030 |

*Notes.* All independent variables are measured at baseline; all dependent variables are measured at follow-up. Values in italics refer to unadjusted analyses without control variables. BPD = Borderline personality disorder; DIKJ = *Depressionsinventar für Kinder und Jugendliche*; DV = dependent variable; IV = independent variable; NSSI = non-suicidal self-injury.

^1^ log-transformed

^2^ After correction for multiple testing using Holm.

**Supplement C**

Post-hoc analyses testing habituation hypothesis: NSSI as a predictor for change in pain sensitivity

|  |  | **Variable** | | | | | |
| --- | --- | --- | --- | --- | --- | --- | --- |
|  |  | NSSI (change or baseline) | Pain threshold/ tolerance | Age | Gender | Medication^1^ | Smoking^1^ |
| Model 1:  DV = Pain threshold,  IV = Change in NSSI frequency | *b* (*SE*) | < 0.01 (0.01); *0.01 (0.01)* | 0.38 (0.08); *0.41 (0.09)* | 0.12 (0.07) | 0.21 (0.28) | 0.35 (0.25) | 0.27 (0.21) |
|  | 95% CI | [-0.02, 0.03]; *[-0.02, 0.03]* | [0.21, 0.55]; *[0.24, 0.58]* | [-0.02, 0.25] | [-0.34, 0.77] | [-0.15, 0.84] | [-0.16, 0.69] |
|  | *t* | 0.42; *0.64* | 4.46; *4.77* | 1.76 | 0.76 | 1.41 | 1.26 |
|  | *p* | .678; .*527* | < .001; *< .001* | .083 | .448 | .163 | .213 |
| Model 2:  DV = Pain tolerance,  IV = Change in NSSI frequency | *b* (*SE*) | 0.01 (0.01); *0.01 (0.01)* | 0.60 (0.12); *0.55 (0.11)* | -0.07 (0.08) | 0.19 (0.31) | 0.44 (0.27) | < -0.01 (0.23) |
|  | 95% CI | [-0.01, 0.04]; *[-0.02, 0.03]* | [0.36, 0.84]; *[0.32, 0.77]* | [-0.23, 0.08] | [-0.42, 0.81] | [-0.10, 0.98] | [-0.47, 0.46] |
|  | *t* | 1.06; *0.67* | 4.95; *4.82* | -0.99 | 0.62 | 1.62 | -0.02 |
|  | *p* | .292; *.508* | < .001; *< .001* | .326 | .536 | .110 | .985 |
| Model 3:  DV = Pain threshold,  IV = NSSI frequency at baseline | *b* (*SE*) | 0.01 (0.01); *0.01 (0.01)* | 0.40 (0.09); *0.41 (0.09)* | 0.10 (0.07) | 0.27 (0.28) | 0.20 (0.24) | -0.08 (0.21) |
|  | 95% CI | [-0.01, 0.03]; *[-0.01, 0.03]* | [0.23, 0.57]; *[0.24, 0.58]* | [-0.03, 0.24] | [-0.28, 0.83] | [-0.27, 0.67] | [-0.50, 0.34] |
|  | *t* | 0.87; *0.87* | 4.67; *4.82* | 1.58 | 0.98 | 0.85 | -0.38 |
|  | *p* | .385; .*385* | < .001; *< .001* | .120 | .329 | .399 | .705 |
| Model 4:  DV = Pain tolerance,  IV = NSSI frequency at baseline | *b* (*SE*) | -0.01 (0.01); *-0.01 (0.01)* | 0.53 (0.12); *0.54 (0.11)* | -0.04 (0.07) | 0.19 (0.31) | 0.17 (0.26) | 0.18 (0.23) |
|  | 95% CI | [-0.03, 0.02]; *[-0.03, 0.02]* | [0.29, 0.76]; *[0.32, 0.76]* | [-0.19, 0.11] | [-0.42, 0.81] | [-0.35, 0.68] | [-0.28, 0.65] |
|  | *t* | -0.59; *-0.72* | 4.48; *4.83* | -0.55 | 0.62 | 0.65 | 0.78 |
|  | *p* | .556; *.476* | < .001; *< .001* | .582 | .538 | .518 | .436 |

*Notes.* All dependent variables are measured at follow-up. The independent variables pain threshold/tolerance, age and gender are measured at baseline for all analyses. The control variables medication and smoking are change variables (difference between follow-up and baseline) for models 1 and 2 and baseline measures for models 3 and 4. Values in italics refer to unadjusted analyses without control variables. *N* = 66. DV = dependent variable; IV = independent variable; NSSI = non-suicidal self-injury.

^1^ Level 1 compared to level 0.
